# The complete mitochondrial genome of *Cacopsyllaburckhardti* (Hemiptera, Psylloidea, Psyllidae)

**DOI:** 10.3897/BDJ.10.e85094

**Published:** 2022-06-08

**Authors:** Euna Jo, Geonho Cho

**Affiliations:** 1 Division of Biotechnology, College of Life Sciences and Biotechnology, Korea University, Seoul, Republic of Korea Division of Biotechnology, College of Life Sciences and Biotechnology, Korea University Seoul Republic of Korea; 2 Division of Life Sciences, Korea Polar Research Institute (KOPRI), Incheon, Republic of Korea Division of Life Sciences, Korea Polar Research Institute (KOPRI) Incheon Republic of Korea; 3 Sunchon National University, Suncheon, Republic of Korea Sunchon National University Suncheon Republic of Korea

**Keywords:** *Cacopsyllaburckhardti*, mitochondrial genome, phylogenetic analysis, pear psyllids, Hemiptera, Psyllidae

## Abstract

*Cacopsyllaburckhardti* Luo, Li, Ma & Cai, 2012 (Hemiptera, Psylloidea, Psyllidae) is a pear psyllid species, distributed in the East Asia. The complete mitogenome of *C.burckhardti* is obtained in this study for the first time. The mitogenome of *C.burckhardti* is circular form and 14,798 bp long, which consists of 13 protein-coding genes, 22 tRNAs and two rRNAs. The base composition is 38.80% for A, 34.89% for T, 9.99% for G and 16.33% for C, with the higher A + T contents (73.69%). The phylogenetic analysis, using 13 protein-coding genes, shows that *C.burckhardti* is clustered with other *Cacopsylla* species and nested in the Psyllidae clade within the superfamily Psylloidea.

## Introduction

Pear psyllids are major pests of cultivated and wild pears. They inflict damage by sucking plant sap, secreting honeydew and vectoring plant diseases on pears (Cho et al. 2017, Cho et al. 2019). *Cacopsyllaburckhardti* Luo, Li, Ma & Cai, 2012 (Hemiptera, Psylloidea, Psyllidae) is a species of pear psyllids, widely distributed in East Asia (i.e. China, Japan, Korea and Far East Russia) (Cho et al. 2017, Cho et al. 2020a). The largest psyllid genus *Cacopsylla* includes hundreds of species and constitutes lots of economically important pest species all around the world; however, there are many taxonomic problems remaining to be solved. Recent molecular phylogenetic studies have been attempted to resolve repeated misidentifications and to reveal their evolutionary relationships using mitochondrial and nuclear genes (Percy et al. 2018, Cho et al. 2019, Cho et al. 2020b, Tsai et al. 2020). To date, only three complete mitochondrial genomes were publicly released in the Genus *Cacopsylla* (Que et al. 2015, Percy et al. 2018, Wang et al. 2021). Here, we report the first complete mitogenome sequence of *C.burckhardti* and its phylogenetic position within the superfamily Psylloidea.

## Material and methods

The samples of *C.burckhardti* were collected from Myeonggae-ri, Nae-myeon, Hongcheon-gun, Gangwon-do, Korea (37°51'37.44"N 128°32'36.72"E). The specimens are deposited in the College of Agriculture and Life Science, Seoul National University (SNU, Seunghwan Lee, seung@snu.ac.kr) under the voucher number 150606GH-30. Total genomic DNA was extracted using Omniprep Genomic DNA isolation kit (G-Biosciences, MO, USA). The quality and quantity of DNA were checked by the gel electrophoresis method and Qubit 2.0 Fluorometer (Life Technologies, CA, USA), respectively. The 150 bp paired-end library was prepared using TruSeq DNA Nano kit (Illumina, CA, USA) and sequenced on an Illumina Novaseq 6000 sequencing system according to the manufacturer’s protocol. A total of 370,678,166 raw reads and 55.972403 gigabases were produced. The average sequencing depth for the mitochondrial genome was 3,718x, which was much higher than the other relatively low depth mitochondrial genomes. FastQC v.0.11.9 (Andrews 2020) was used to check the quality and to filter out the adapters and low-quality reads. *De novo* assembly of mitochondrial genome of *C.burckhardti* was conducted using 363,443,312 trimmed reads and GetOrganelle pipeline (Jin et al. 2020). The genes were annotated through MITOS web server (Bernt et al. 2013), followed by manual curation using Geneious 6.1.7 (Kearse et al. 2012). A circular map of the mitochondria was generated using OGDRAW v.1.3.1 (Lohse et al. 2007).

## Results and Discussion

**Figure 1.**
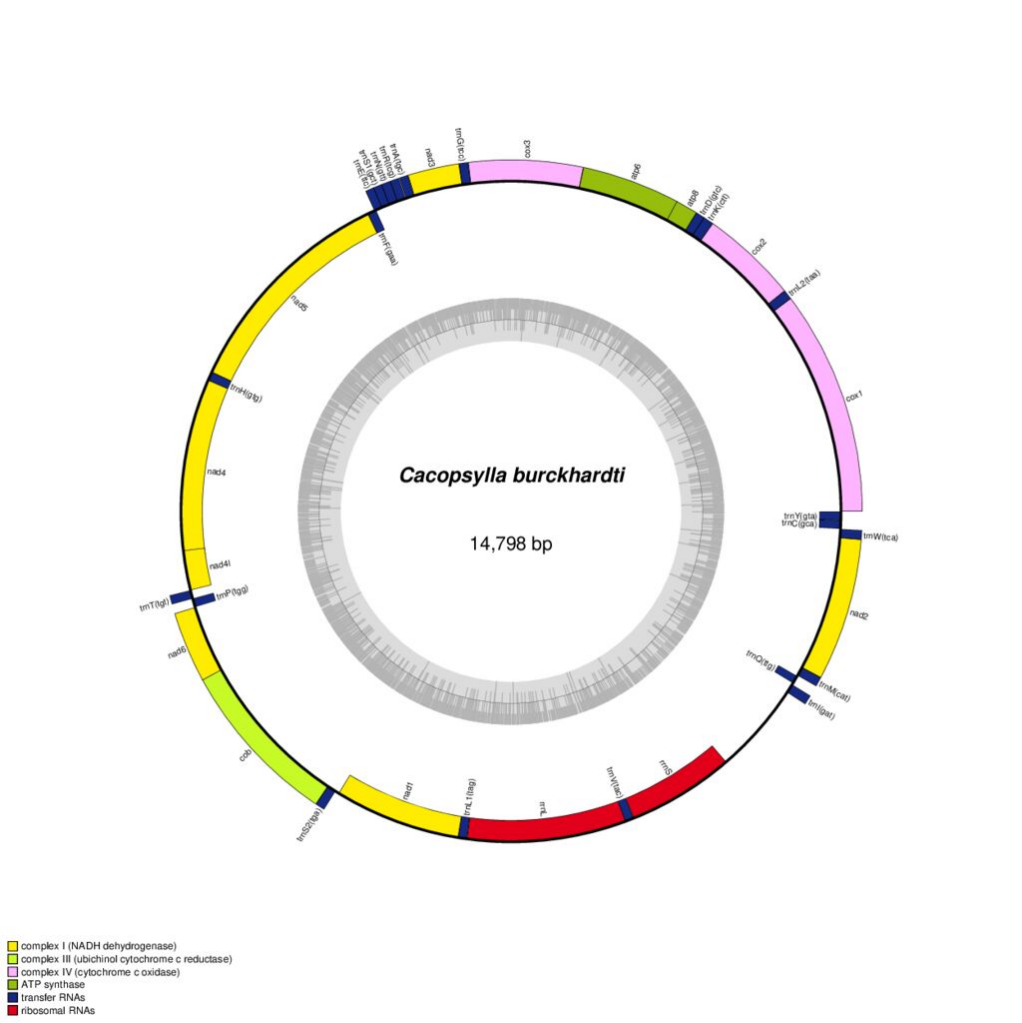
Complete mitochondrial genome map of *Cacopsyllaburckhardti*. The grey small circle represents GC content graph.

**Figure 2.**
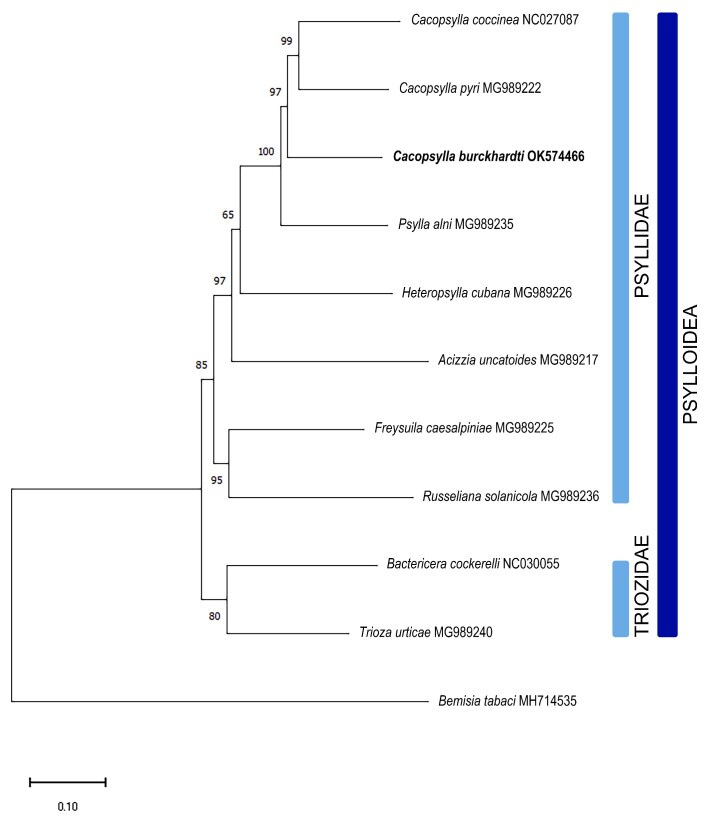
Phylogenetic relationship between *Cacopsyllaburckhardti* and other psylloid species, based on 13 protein-coding genes of mitochondrial genomes. The tree was constructed using the neighbor-joining (NJ) method with 10,000 bootstrap replicates. The bootstrap support values are shown on each node. The scientific names and GenBank accession numbers are shown for each branch.

The complete mitochondrial genome of *C.burckhardti* (GenBank accession no. OK574466) is 14,798 bp in length, including 13 protein-coding genes, 22 transfer RNA genes and two ribosomal RNA genes (Fig. 1). The base composition is A (38.80%), T (34.89%), G (9.99%) and C (16.33%), with a high A + T contents of 73.69%. The protein-coding genes have four types of start codons (6 ATAs, 4 ATGs, 2 TTGs and 1 ATT) and three types of stop codons (9 TAAs, 1 TAG and 3 Ts). Based on the 13 protein-coding genes, phylogenetic relationships were analysed for 10 species from the superfamily Psylloidea, with *Bemisiatabaci* (MH714535) as outgroup (Fig. 2). The neighbor-joining (NJ) method with 10,000 bootstrap replications was implemented by MEGA11 software (Tamura et al. 2021). The tree shows that *C.burckhardti* is clustered with *Cacopsylla* species (*C.coccinea* and *C.pyri*) and they are grouped with other species belonging to Psyllidae (*Psyllaalni*, *Heteropsyllacubana*, *Acizziauncatoides*, *Freysuilacaesalpiniae* and *Russellianasolanicola*). The mitogenome of *C.burckhardti* will be an important addition to explore the evolutionary relationships of pear psyllids as well as the superfamily Psylloidea.
